# Emerging Infectious Disease Leads to Rapid Population Declines of Common British Birds

**DOI:** 10.1371/journal.pone.0012215

**Published:** 2010-08-18

**Authors:** Robert A. Robinson, Becki Lawson, Mike P. Toms, Kirsi M. Peck, James K. Kirkwood, Julian Chantrey, Innes R. Clatworthy, Andy D. Evans, Laura A. Hughes, Oliver C. Hutchinson, Shinto K. John, Tom W. Pennycott, Matthew W. Perkins, Peter S. Rowley, Vic R. Simpson, Kevin M. Tyler, Andrew A. Cunningham

**Affiliations:** 1 British Trust for Ornithology, Thetford, Norfolk, United Kingdom; 2 Institute of Zoology, Zoological Society of London, London, United Kingdom; 3 The Royal Society for the Protection of Birds, Sandy, United Kingdom; 4 Universities Federation for Animal Welfare, Wheathampstead, United Kingdom; 5 Department of Veterinary Pathology, University of Liverpool, South Wirral, United Kingdom; 6 Electron Microscopy Unit, UCL Medical School, London, United Kingdom; 7 Disease Surveillance Centre, Scottish Agricultural College, Ayr, United Kingdom; 8 Wildlife Veterinary Investigation Centre, Truro, United Kingdom; 9 Biomedical Research Centre, School of Medicine, Health Policy and Practice, University of East Anglia, Norwich, United Kingdom; University of Bristol, United Kingdom

## Abstract

Emerging infectious diseases are increasingly cited as threats to wildlife, livestock and humans alike. They can threaten geographically isolated or critically endangered wildlife populations; however, relatively few studies have clearly demonstrated the extent to which emerging diseases can impact populations of common wildlife species. Here, we report the impact of an emerging protozoal disease on British populations of greenfinch *Carduelis chloris* and chaffinch *Fringilla coelebs*, two of the most common birds in Britain. Morphological and molecular analyses showed this to be due to *Trichomonas gallinae*. Trichomonosis emerged as a novel fatal disease of finches in Britain in 2005 and rapidly became epidemic within greenfinch, and to a lesser extent chaffinch, populations in 2006. By 2007, breeding populations of greenfinches and chaffinches in the geographic region of highest disease incidence had decreased by 35% and 21% respectively, representing mortality in excess of half a million birds. In contrast, declines were less pronounced or absent in these species in regions where the disease was found in intermediate or low incidence. Also, populations of dunnock *Prunella modularis*, which similarly feeds in gardens, but in which *T. gallinae* was rarely recorded, did not decline. This is the first trichomonosis epidemic reported in the scientific literature to negatively impact populations of free-ranging non-columbiform species, and such levels of mortality and decline due to an emerging infectious disease are unprecedented in British wild bird populations. This disease emergence event demonstrates the potential for a protozoan parasite to jump avian host taxonomic groups with dramatic effect over a short time period.

## Introduction

Emerging infectious diseases (EIDs) are increasingly cited as threats to wildlife, livestock and humans alike [1] and can be a major threat to geographically isolated or critically endangered wild bird populations [2], [3]. Parasites are integral components of healthy ecosystems, but while impacts on individuals are well recognised [e.g. 4,5] consequences at the population level are poorly understood. Assessing the population impacts of disease, particularly those caused by emerging pathogens, within wildlife populations is problematic because little is known of the background species complement of their parasites and because the detection and diagnosis of disease in most wildlife species is challenging. Also, there usually is a paucity of host population data before and after disease emergence. Consequently, documented population declines of common or widespread avian populations due to infectious disease are rare [6], . Here we combine systematic large-scale monitoring schemes to quantify the incidence of an emerging disease in three widespread passerine bird species and its population impacts.

*Trichomonas gallinae* is a common protozoan parasite of pigeons (Columbiformes) which principally infects the upper alimentary tract where it can cause the disease, necrotic ingluvitis [9]. Epizootic mortality in columbiform species has been previously reported [9] and the parasite infrequently infects other avian taxa such as birds of prey and songbirds [10], [11]. Trichomonosis has been postulated to be a factor contributing to the extinction of the passenger pigeon *Ectopistes migratorius* [12] and has been shown to be a significant cause of nestling mortality in the island-endemic pink pigeon *Nesoenas mayeri* [3] and in the Iberian Peninsula population of the Bonelli's eagle *Hieraaetus fasciatus* [13], [14]. Both of these small populations are considered to be endangered and, for both, mortality due to trichomonosis has been highlighted as a conservation concern.

**Figure 1:**
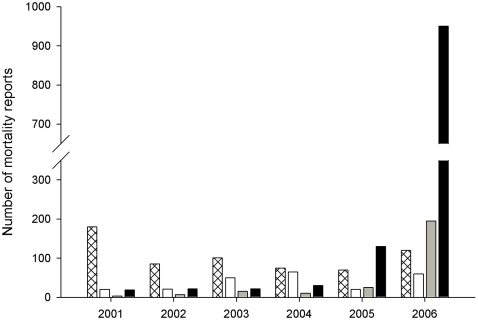
Seasonal incidence of opportunistic reports in all garden bird mortality 2001–2006. Hatched bars – winter (Dec-Feb), open bars – spring (March–May), stippled bars – summer (June–Aug) and black bars - autumn (Sept-Nov). Note break in axis indicating an unprecedented level of reporting in autumn 2006.

Opportunistic surveillance of bird deaths in Britain since 2000 has shown a seasonal pattern in finch mortality driven primarily by low-levels of salmonellosis (Fig. 1, [15]). In autumn 2005, the number of unsolicited reports of bird mortality increased markedly and early investigations identified infection with a trichomonad parasite [16], [17]. The geographic spread of these reports was uneven and here we take advantage of long-term volunteer monitoring of garden bird occurrence to (i) quantify disease incidence in three species of common garden bird: greenfinch *Carduelis chloris*, chaffinch *Fringilla coelebs* and dunnock *Prunella modularis*; (ii) demonstrate a spatially contemporaneous decline in the occurrence of frequently affected bird species in gardens; and (iii) combine this with national monitoring of bird abundance to show that this decline was followed by significant reductions in regional breeding populations. Public reporting of wild bird carcasses has been utilised as a surveillance tool for West Nile virus and Usutu virus elsewhere [18], [19] and volunteer networks were successfully instituted in North America to elicit reports of diseased birds in order to characterise the spread of mycoplasmal conjunctivitis in the house finch *Carpodacus mexicanus* [20], [21]. This is the first time, however, as far as we are aware, that such quantitative monitoring of disease incidence and its population impact has been undertaken using established survey networks.

## Results

### Identification of the disease epidemic

Opportunistic monitoring of garden bird mortality by the Royal Society for the Protection of Birds (RSPB) between 2001 and 2004 showed an annual seasonal peak in mid-winter (Dec/Jan), with 37–76% of reports per annum occurring in these two months (Fig. 1, K. Peck, *Unpublished data*); post mortem examinations indicated that this seasonal peak was largely due to salmonellosis in Fringillidae and Passeridae species ([15] Kirkwood, Cunningham and Simpson, *Unpublished data*). Between January 1^st^ 2000 and December 31^st^ 2004, we examined 750 garden birds post mortem, of which 67% of greenfinch deaths (168/252 birds) were due to salmonellosis and no cases of finch trichomonosis were confirmed.

Following the index case of trichomonosis in a British finch in April 2005, small numbers of finch mortality incidents were reported throughout 2005, unusually peaking during September to November [17]. In summer 2006, the number of finch, particularly greenfinch, mortality reports increased dramatically with a total of 1054 trichomonosis incidents recorded (according to our incident definition – see below), involving c. 6300 dead greenfinches and chaffinches combined, between 1 April 2006 and 30 September 2006. This comprised 50% of all reported incidents of garden bird morbidity and mortality during this period and compares with 84 incidents of trichomonosis for the same time period in 2005 and sporadic cases (none of which involved finches) in previous years. These reports from the public were unsolicited, not in response to a direct appeal and occurred prior to media coverage of the EID. Sick and dead birds at affected sites were typically observed in close vicinity to garden bird feeding stations and exhibited non-specific signs of malaise, for example lethargy and fluffed-up plumage, frequently in combination with dysphagia.

### Identification of the disease organism

**Figure 2:**
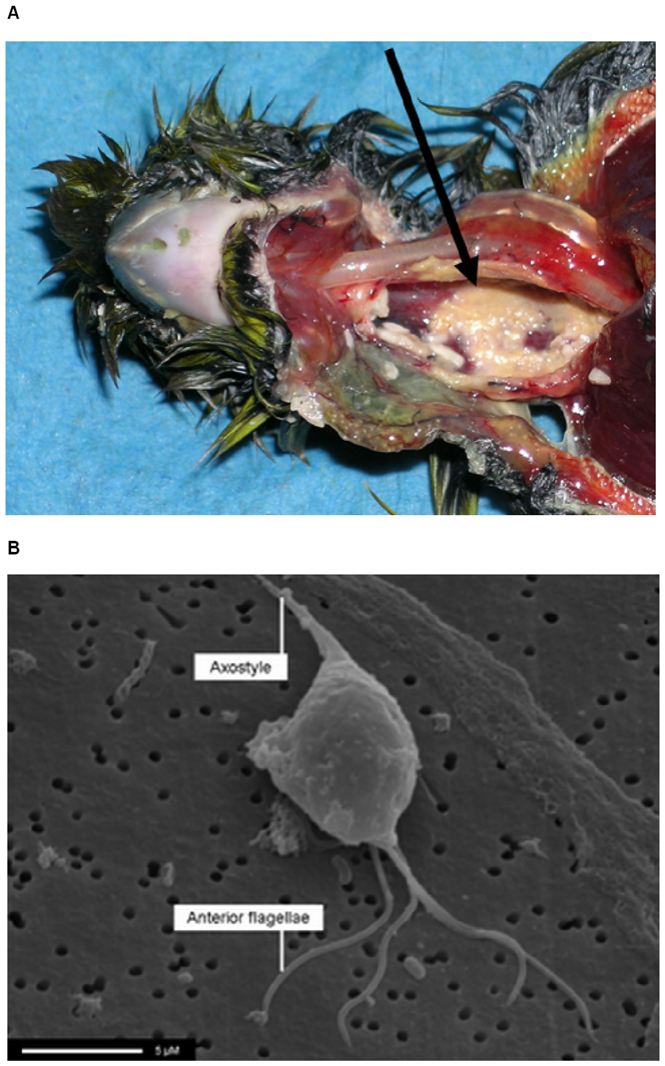
Necrotic ingluvitis lesions and trichomonad parasite morphology. (a) Necrotic ingluvitis lesions (arrow) with a characteristic yellow caseous appearance in a greenfinch caused by *Trichomonas gallinae* infection. (b) Morphology of the greenfinch trichomonad parasite. Scanning electron micrograph. Arrows indicate anterior flagella and axostyle.

Necrotic ingluvitis, typically extending through the full thickness of the oesophageal wall and often involving adjacent connective tissue, was diagnosed through post-mortem examination (Fig. 2a) and confirmed as trichomonosis, according to our case definition (see below), in 70 of 125 greenfinches, and in 18 of 76 chaffinches examined between 1 April 2006 and 30 September 2006. These diagnoses were reached on the basis of *T. gallinae* culture alone in 17 birds, nested PCR amplification alone in 58 birds, and a combination of parasite culture and nested PCR in 13 birds. All confirmed trichomonosis cases were negative for *Salmonella* sp. on culture. In addition, 20 of the 125 greenfinches and 29 of the 76 chaffinches were suspected to have died of trichomonosis as these birds had necrotic ingluvitis which was negative for *Salmonella* sp. Trichomonosis in these cases, however, was not confirmed using nested PCR or culture.

Of 241 finch carcasses examined during the period 1 April 2006 to 30 September 2006, 179 were diagnosed as having died of infectious disease. Of these 179 birds, trichomonosis accounted for 144 (80%) of the deaths (90 greenfinches, 47 chaffinches and 7 birds from four other finch species), *E.coli* serotype O86 (the second most common infectious cause of death and which is associated with enteritis with no necrotic ingluvitis) was diagnosed in 24 (13%), while salmonellosis was confirmed in only 4 (2%).

Fringillidae species accounted for 84% (292/347) of trichomonosis cases diagnosed in 2005 and 2006, including greenfinch (173 cases), chaffinch (106 cases) and four other finch species (5 bullfinch *Pyrrhula pyrrhula*, 4 goldfinch *Carduelis carduelis*, 2 brambling *Fringilla montifringilla* and 2 siskin *Carduelis spinus*). Columbidae species accounted for 11% (37/347) of cases (20 collared dove *Streptopelia decaocto* and 17 wood pigeon *Columba palumbus*). The only other species in which the disease was diagnosed were house sparrow *Passer domesticus* (9 cases), yellowhammer *Emberiza citrinella* (4 cases), dunnock (3 cases) and great tit *Parus major* (2 cases). Concurrent soiling of the beak and facial plumage with food and saliva was frequently present in affected finches and such birds were typically thin or emaciated. Histopathological examination of the crop confirmed focally extensive moderate to severe mucosal ulceration and submucosal necrosis with infiltration by moderate numbers of degenerate and viable heterophils, lymphocytes and macrophages. Superficially, there was often a layer of necrotic crop epithelial tissue within which groups of 10–20 µm diameter round cells (consistent with protozoal organisms) and numerous clusters of mixed bacterial colonies were seen. Autolysis of the alimentary tract precluded meaningful histological examination in many cases; consequently histopathology was not used as a routine diagnostic test for confirmation of trichomonosis.

Giemsa-stained parasite culture preparations revealed a variable morphology (body dimensions range 8–11×4–5 µm) typical of a trichomonad parasite with a single nucleus and axostyle, anterior flagella and an undulating membrane. Scanning and transmission electron microscopy (Fig. 2b) confirmed the presence of a parasite with plastic pyriform morphology and four anterior flagella that typically exited the body together in pairs. A prominent undulating membrane, with no free posterior trailing flagellum, was present.

**Figure 3:**
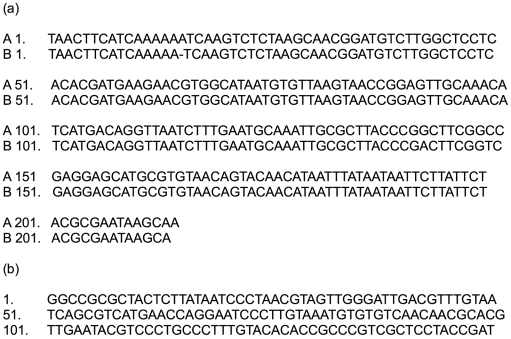
Sequence data from British finch trichomonad samples. (a) Nucleotide sequence (214 nucleotides) from amplification and sequencing of the ITS1/5.8S/ITS2 ribosomal region using TFR1 and TFR2 primers from (A) Consensus sequence from British finch (Genbank GQ150752 and GQ150753) trichomonad samples and (B) *Trichomonas gallinae* (Rivolta) Stabler (ATCC® Number 30230 TM). (b) Nucleotide sequence (149 nucleotides) from nested PCR with trichomonad SSU rRNA primers followed by TN3 and TN4 nested primers (Genbank GQ214405).

Amplification of the ITS1/5.8S/ITS2 ribosomal region was performed on DNA extracted from oesophageal lesions from nine greenfinches and nine chaffinches (submitted from 18 disparate sites across 13 counties covering England, Wales and Scotland) that died of trichomonosis in 2005 or 2006. An identical consensus sequence of 214 nucleotides was identified for all PCR products (Fig. 3a, Genbank accession numbers GQ150752 and GQ150753) from the finch samples examined. National Centre for Biotechnology Information (NCBI) BLAST search identified that the consensus finch sequence matched four Genbank entries with100% sequence identity with 100% query coverage, all of which were for *Trichomonas gallinae* (EU215369 (multiple columbid and hawk species from the USA), EU290649 (house finch *Carpodacus mexicanus*/corvid species from the USA), EF208019 (Mauritian columbid species), AY349182 (*T. gallinae* strain g7)). Thus the organism infecting the British finches was identified as *T. gallinae*.

Sequencing of second stage products of a nested PCR for the detection of trichomonads from oesophageal lesion extracts from seven greenfinches and one chaffinch examined in 2005 or 2006 with trichomonosis identified a consensus sequence of 149 nucleotides in all eight cases (Fig. 3b, Genbank accession no GQ214405). NCBI BLAST identified the consensus finch sequence as a match for four *T. gallinae* Genbank entries (EU215372.1 (Cooper's hawk *Accipiter cooperii*), EU215373.1 (rock pigeon *Columba livia*), EU215374.1 (collared dove), and EU215375.1 (broad-winged hawk *Buteo platypterus*); all from the USA) with 100% sequence identity and 100% query coverage and five *Trichomonas* sp. reports with 100% sequence identity and 97% query coverage. Multiple *Tetratrichomonas gallinarum* and *Tetratrichomonas* sp. reports had 98% or less sequence identity with 100% query coverage. The specificity of the nested PCR for a range of species within the Trichomonadidae was not assessed in this study, therefore this technique cannot currently be used in isolation as a diagnostic test for *T. gallinae*. However, use of the nested PCR appears robust within the case definition for finch trichomonosis that we have employed.

### Geographical and temporal distribution of the epidemic

**Figure 4:**
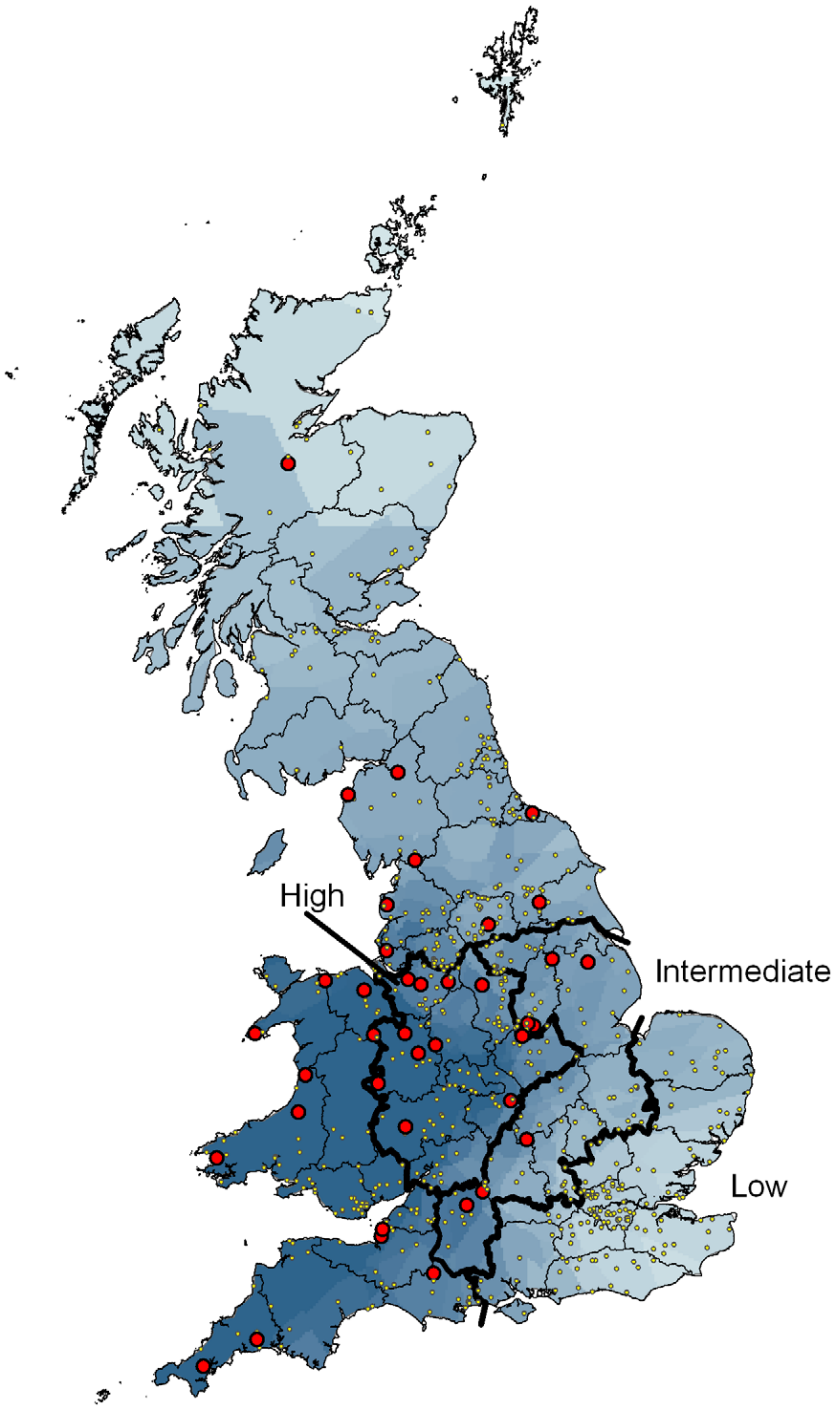
Distribution of finch trichomonosis incidents in 2006. Gardens reporting at least one incident of finch trichomonosis (large red dots) and all other sites (small yellow dots) contributing to the systematic survey. The shading indicates relative incidence of trichomonosis recorded by the opportunistic survey (incidents per thousand households for each county interpolated from county centroids). The heavy lines delineate areas of High, Intermediate and Low incidence, based on the opportunistic survey data.

Rates of opportunistic reports of trichomonosis (identified according to our incident definition) varied greatly among counties, with rates in excess of 0.20 incidents per thousand households (ipth) found in Gloucestershire, Powys and Warwickshire. We used the opportunistic reports to define regions of High, Intermediate and Low disease incidence (Fig. 4). Overall, the average reporting rate was 0.037 ipth but this varied markedly among the regions (aggregate average for the High region: 0.109 ipth; Intermediate region: 0.056 ipth; Low region: 0.003 ipth, χ^2^ = 650.5, d.f. = 2, *p*<0.001). As these rates may have been influenced by local publicity, we quantified incidence in a network of garden sites where diseased birds were searched for systematically according to a defined protocol throughout the study period. Trichomonosis (according to our incident definition) was identified at 39 sites (5.2%) but the incidence varied spatially in a manner similar to the opportunistic reports, with 10.4% (n = 115) of participants in the High region reporting an incident, 7.8% (n = 154) in the Intermediate region and 0.0% (n = 164) in the Low region (χ^2^ = 16.4, d.f. = 2, *p*<0.005).

### Changes in bird abundance

**Figure 5:**
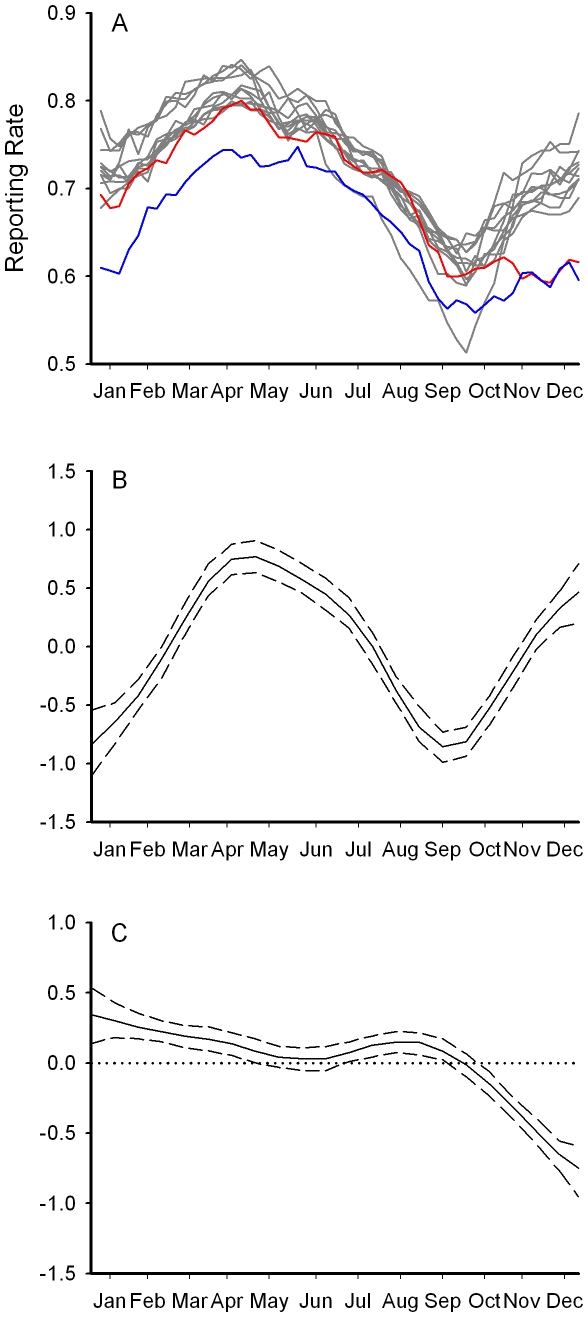
Seasonal variation in greenfinch occurrence in gardens. (a) Reporting rate for greenfinch in all GBW gardens for the years 1996–2005 (grey lines), 2006 (red) and 2007 (blue). (b) Fitted seasonal pattern of mean peak greenfinch count in 828 GBW gardens with complete counts in 2005. (c) Difference in mean peak count throughout the year between 2005 and 2006 for greenfinch, dashed lines represent 95% confidence limits.

The weekly reporting rates of greenfinch occurrence in all gardens contributing to the British Trust for Ornithology's (BTO) Garden BirdWatch survey [22] show a seasonal pattern, with more gardens reporting birds in spring and fewer in the autumn (Fig. 5a). There was a significant difference in seasonal pattern of occurrence between 2005 (which was very similar to previous years, Fig. 5b) and 2006, with markedly fewer gardens reporting greenfinches from early August (week 32) onwards (Fig. 5c, F_9,23_ = 24.23, p<0.0001). Analysis of a subset of these sites which recorded counts of individual birds (rather than presence) showed a similar reduction in mean abundance in gardens reporting greenfinches in the latter half of 2006.

**Figure 6:**
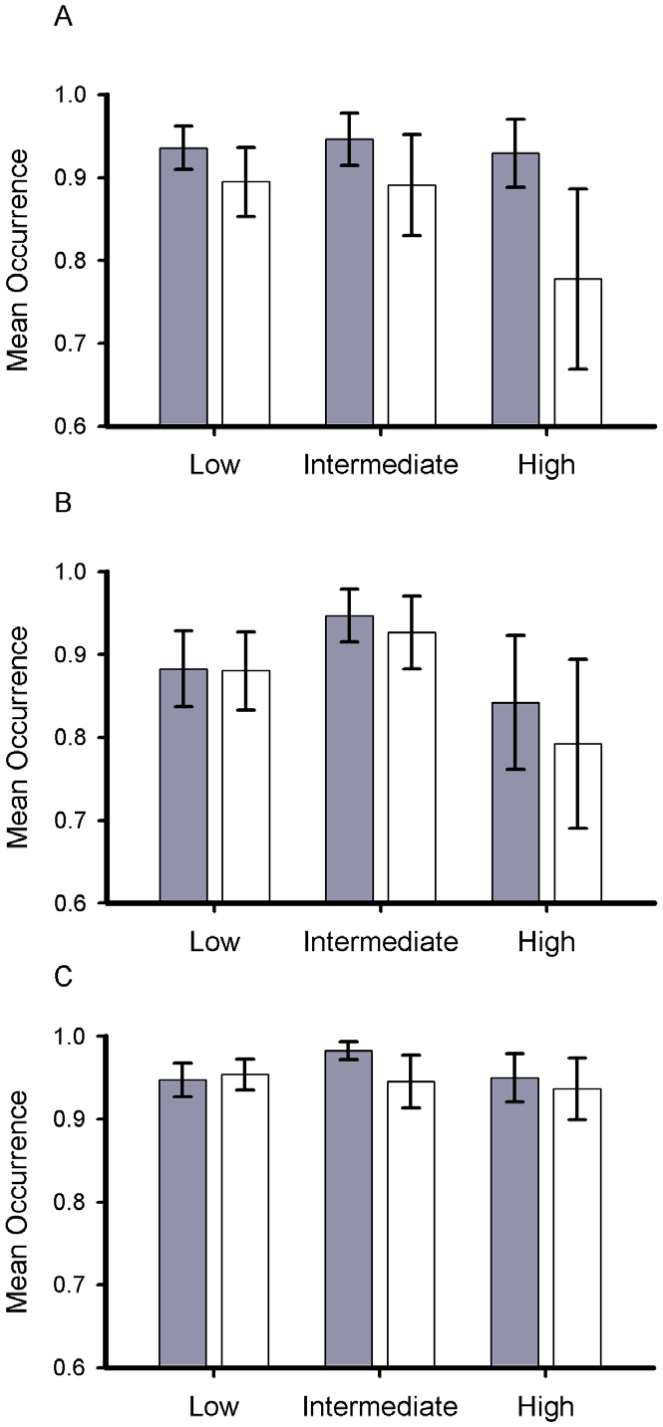
Regional change in greenfinch occurrence in gardens in response to trichomonosis. Mean reporting rate from GBW of greenfinch, chaffinch and dunnock in spring 2005/06 (filled bars) and 2007 (open bars) in areas of Low, Intermediate and High incidence of trichomonosis incidence (see Fig. 3). Bars represent 95% confidence limits.

The reporting rate for greenfinches in the following spring (2007) was significantly reduced in the area of High trichomonosis-associated mortality (*β* = −1.32±0.12, *p*<0.001), but less so in the region with Intermediate (*β* = −0.77±0.15, *p*<0.001) or Low mortality levels (*β* = −0.53±0.08, *p*<0.01) (Fig. 6). Reductions in occurrence of chaffinch (*β* = −0.53±0.12, *p* = 0.02) and dunnock (*β* = −0.25±0.12, *p* = 0.04) in the region of High trichomonosis-associated mortality were lower, and there were no significant reductions in occurrence of either species in the regions of Intermediate or Low incidence.

**Figure 7:**
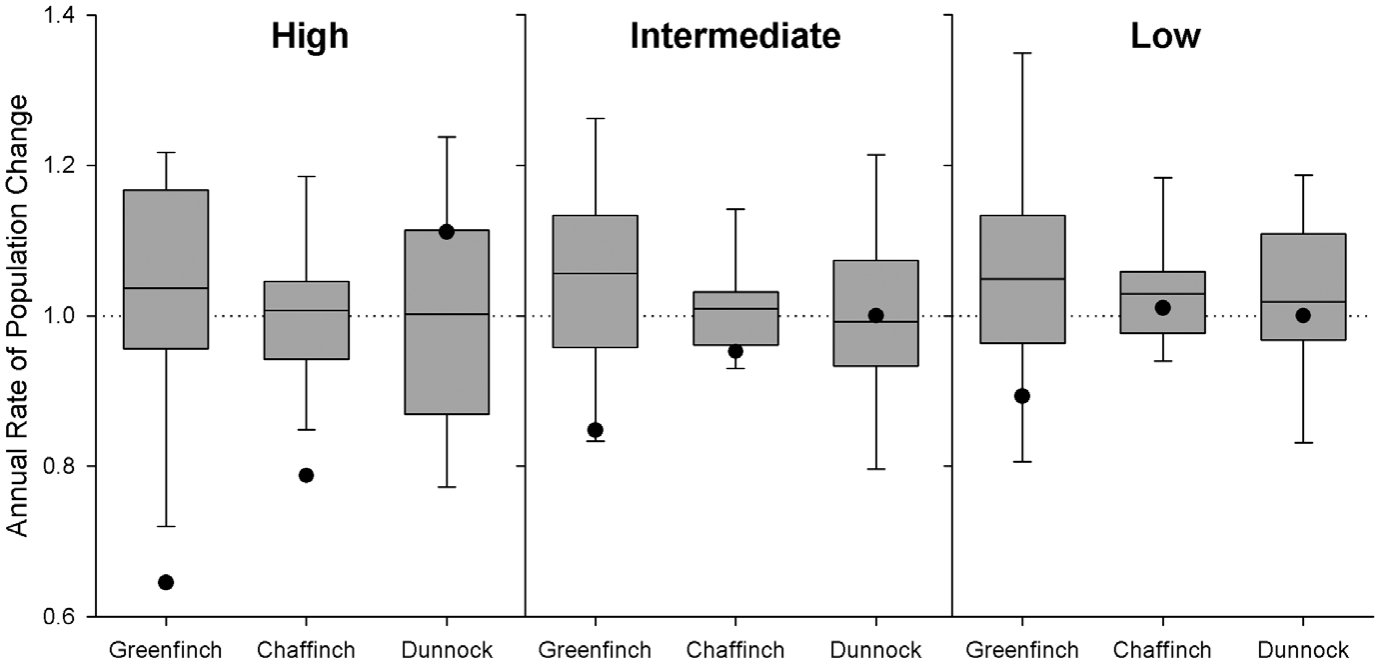
Annual rate of population change as measured by the BBS in areas of differing disease incidence. Boxes show mean and quartiles of annual changes and whiskers minimum and maximum annual change observed in the period 1994–2006; points, the population change recorded in 2007. Dotted line indicates no population change.

These observed reductions in occurrence were reflected in changes in the size of wider regional breeding populations obtained from the independently-derived Breeding Bird Survey (BBS) [23]. The decline in relative abundance of greenfinches on BBS squares was significantly greater (35%) in the region of High trichomonosis-associated mortality than in either of the other two regions (Table 1). This annual change was much more marked than any seen in the previous ten years (Fig. 7). In accordance with our predictions, the number of breeding chaffinches declined significantly (21%) in the High incidence region but not in the other two areas; abundance of breeding dunnocks did not decline in any region.

**Table 1:** Population change of breeding birds between 2006 and 2007.

	Low		Intermediate		High	
	n	Change	n	Change	n	Change
Greenfinch	477	−10.9 (−17.3, −4.1)	433	−15.2 (−21.8, −8.1)	232	−35.5 (−42.3, −27.9)
Chaffinch	549	+0.6 (−3.7, +5.1)	526	−4.4 (−8.7, +0.2)	256	−21.3 (−25.7, −16.5)
Dunnock	496	−1.2 (−7.0, +7.3)	465	−0.3 (−7.7, +7.6)	286	+11.4 (+1.7, +22.1)

Population change between 2006 and 2007 in areas of High, Intermediate and Low incidence of trichomonosis mortality during autumn 2006 derived from the Breeding Bird Survey. The percentage change between the two years is given with approximate 95% confidence limits, n is the number of BBS squares in each region in which the species was recorded.

## Discussion

We diagnosed trichomonosis as an emerging and widespread cause of death in British finches in 2005 and 2006. The gross and histopathological findings are consistent with upper alimentary tract lesions caused by *T. gallinae* infection in columbiform species [24], [25], although lesions in finches typically occur in the proximal oesophagus, compared to the pharyngeal region in affected pigeons and doves. Ultrastructural examination identified morphology consistent with *Trichomonas* sp. protists, including *T. gallinae* [26]–[28]. Amplification and sequencing of the ITS1/5.8S/ITS2 region using TFR1 and TFR2 primers yielded consensus sequence with 100% identity to published accounts for *T. gallinae*, confirming the parasite species identification [10], [29]–[31].

Experimental assessment of the survival of *T. gallinae* in white-winged dove *Zenaida asiatica* carcasses found that most reliable diagnostic results were obtained on sampling within 8 hours of death and that parasite culture from *T. gallinae*-positive birds was successful in only 44% of carcasses sampled at 48 hours following death [32]. In the current study, parasite culture was found to be a useful technique for confirmation of the diagnosis in carcasses even in a mild state of autolysis. Due to delays in submission, however, negative culture results could not be used to exclude the diagnosis. Therefore, nested PCR provided a useful ancillary diagnostic tool in combination with post mortem and microbiological examinations within our case definition for the diagnosis of trichomonosis for carcasses in which the protozoan parasites were no longer viable.

Consideration of two independent data sets of bird mortality in Britain (i.e. (1) surveillance based opportunistically on unsolicited reports from members of the public and (2) systematic monitoring according to a defined protocol by our volunteer network) in combination enables us to maximise their relative benefits. The large-scale, but ad hoc, sampling from the opportunistic survey provided indication of the commencement of the epidemic and samples to determine its epidemiology. The systematic sampling using a pre-existing network provided a quantitative measure of incidence which will be relatively robust to, for example, increases in reporting frequency in response to media coverage. Such data could not realistically be collected in any other way and should, over time, provide much needed detail on the background complement of disease agents in wild bird populations.

The winter of 2006 was relatively mild (mean temperature anomaly in England for Nov-Mar +1.7°C [33]) so mortality levels would be expected to be low [34]. Also, the number of birds, especially seed-eaters, recorded in gardens would be expected to be lower than usual due to a reduced reliance on provisioning [35] and a small reduction in the reporting rate of dunnock was observed in the area of high disease incidence perhaps for this reason. However, the much larger reduction in the occurrence of greenfinches in gardens was not simply because birds were not coming into gardens for food, since reporting continued to be low into the following spring (Fig. 5) and regional declines in greenfinch breeding populations were observed in an independent dataset (BBS) with broader habitat coverage. The onset of the decline in greenfinch reporting rate is contemporaneous with the onset of the trichomonosis outbreak (Fig. 5) and the spatial pattern in regional population decline (Table 1) matches that of the disease occurrence (Fig. 4). We have been unable to identify any other factor that could have caused such large-scale mortality. Furthermore, there were no similarly large declines noted in any other garden bird species (Toms, *unpublished data*).

In Great Britain as a whole, the greenfinch breeding population increased steadily (by c. 60%) from the mid 1980s to 2006, but decreased (significantly) by 15% in 2007 compared to the previous year [36]. It seems likely that this decline was driven, in large part, by the emergence of trichomonosis. Given that the population of greenfinches in Great Britain is in the order of 4 million [23], this represents mortality of a very large number of individuals, possibly in the order of half a million birds. This level of mortality due to an EID is unprecedented in British wild bird populations and is of international interest as, although other studies have demonstrated trichomonosis in non-columbiform species [10], this is the first trichomonosis epidemic to show substantial negative impact on free-ranging populations of these species. The current study also reinforces the value of large-scale, long-term, citizen science programmes for wildlife population monitoring and disease surveillance and of a multi-disciplinary approach to investigating the conservation significance of wildlife disease. As the opportunistic reports from the public were unsolicited, rather than in response to a direct appeal, such as the one undertaken to monitor the spread of house finch mycoplasmosis in the USA [20], we predict large-scale under-reporting of the 2006 trichomonosis epidemic. Our use of volunteers to actively search for sick and dead birds in a systematic fashion, however, should provide a reliable estimate of relative disease incidence (with up to 10% of monitored gardens recording at least one incident of morbidity or mortality) and this was temporo-spatially consistent with the observed population declines in greenfinch and chaffinch populations.

The origin of *T. gallinae* infection in finches is currently unknown but columbiform species are considered the most likely source. Future research priorities include molecular studies to identify the origin of *T. gallinae* in finches, for example through comparing parasite isolates derived from finch, columbiform and other wild bird species, and to assess *T. gallinae* strain diversity in Great Britain on the basis of geography, species and virulence. Retrospective surveys using the nested PCR methodology on archived garden bird tissues, collected prior to 2005, could identify presence of the parasite in non-columbiform populations prior to the onset of epidemic finch mortality, although the chance of detecting a low prevalence from the relatively small number of archived carcasses would be low.

Although greenfinches appear to be the species most frequently affected by *T. gallinae* infection in garden habitats (and chaffinches somewhat less so), the reasons for this are not clear. The greenfinch is one of the species most frequently affected by other infectious diseases that are commonly diagnosed in garden birds, such as salmonellosis and colibacillosis (Lawson and Cunningham, *unpublished data*). The gregarious and granivorous habits of finch species sharing food and water at feeding stations with high contact rates are likely to facilitate pathogen spread. Trichomonosis, however, was confirmed only rarely in members of the Paridae, which also commonly flock to garden bird feeders, so feeding behaviour is unlikely to be the sole driver of greenfinch susceptibility. Investigations of wildlife species mortality which rely on the reporting of sick and dead birds by members of the public have an inherent risk of bias due to variation in observer effort. Also, some species, such as large or brightly coloured ones, are more likely to be detected. Systematic sampling by volunteers in our network provides a relatively consistent level of observer effort and will have reduced variability in detection bias between gardens so should provide a robust measure of relative disease incidence. Although we have quantified the occurrence of trichomonosis in dead birds, the overall prevalence of *T. gallinae* infection in wild bird populations remains unknown. Prospective studies to screen multiple species of live birds for trichomonad parasites [37] would help address this knowledge gap. Experimental challenge studies in multiple species are required to definitively confirm the extent of interspecific variation in susceptibility to infection with, and to disease caused by, *T. gallinae*.

Land-use change and habitat degradation have led to an increased national focus on garden habitats as a useful refuge for British wildlife [38]. It has been estimated that 48% of gardens in Britain provide some form of artificial food for wildlife [39]. Anthropogenic provisioning of wild birds in garden habitats influences contact rates among conspecifics and alters species complements at feeding sites; both factors influence pathogen transmission and exposure rates [40]. Garden bird feeding practice in Great Britain has altered over recent years with increased adoption of summer feeding and increased provision of sunflower and niger seed which might have led to increased concentration of birds at feeders. *Trichomonas gallinae* can be transmitted through direct contact between birds, for example courtship and feeding of young, and through indirect routes including shared food and water sources [9], [41], however further studies are required to assess the relative importance of these transmission routes for finches. Establishing the nature and frequency of disease transmission at garden feeders is thus clearly important to identify if mitigation measures are required and, if so, how they should be employed. The greenfinch and chaffinch are both common garden bird visitors in England and Wales across the year, ranking number 9 and 10 in the most frequent garden visitors in the GBW scheme [22]. Both species are gregarious, visiting gardens in flocks, but other granivorous passerine species are reported in a comparable number of gardens around feeding stations, for example house sparrows, great tits and blue tits, and they also feed in groups. More generally, *T. gallinae* is a pathogen of potential significance to the racing pigeon, aviculture, game bird and poultry industries and the implications for finch trichomonosis to these industries remain poorly understood. Continued monitoring of diseases in wild bird populations is required to better quantify and understand their impact on population dynamics [6], [42] and to identify future changes in host-parasite relationships.

## Materials and Methods

### Ethics statement

No live animals were used for this research, however, the project was reviewed and approved by the Zoological Society of London's Ethics Committee

### Identification of the disease epidemic

Since 2000, opportunistic nationwide monitoring of the causes of garden bird mortality in Great Britain has been carried out by the Institute of Zoology (IoZ, London), the Wildlife Veterinary Investigation Laboratory (Cornwall) and the Scottish Agricultural Colleges (Ayrshire). In 2005, these organisations, together with the BTO, the RSPB, the Department of Veterinary Pathology, University of Liverpool and the Universities Federation for Animal Welfare, established a coordinated surveillance network as part of the Garden Bird Health initiative (GBHi). The GBHi was established before the emergence of trichomonosis in British finches was identified and has always included wide-ranging investigations to identify pathogens responsible for garden bird disease.

The GBHi surveillance of garden bird morbidity and mortality takes place via opportunistic reports obtained from the general public and weekly reports from identified volunteers who form a systematic reporting network. As opportunistic reports are vulnerable to temporal or spatial observer bias, for example following regional media reports, the systematic surveillance network was established in April 2005 in order to quantify disease prevalence rates. This network utilised the BTO's Garden BirdWatch (GBW) volunteer network of approximately 15,000 households throughout Britain [22]. A random sample of 1,614 volunteers (stratified by the number of participants in each recording region) were approached with a view to recording additional information relating to the incidence of diseased and dying birds in their gardens. Of those GBW volunteers approached, 752 submitted data to the GBHi project in 2006. A visual examination of the spatial distribution of sites suggested no spatial bias in those who submitted data relative to those initially approached and there was no difference in the distribution of latitudes (F1,2356 = 1.14, NS) or longitudes (F1,2356 = 1.31, NS) between the two samples.

### Identification of disease organism

Post mortem examinations using a standardised protocol were performed on carcasses submitted from a subset of reported garden bird mortality incidents. Cases were selected for post-mortem investigation based on fresh carcass availability. Birds thought to have died as a result of trauma, predation or infectious disease were examined; our selection criteria did not specifically or solely target finch species or suspected cases of trichomonosis, but rather aimed to achieve a representative cross-section of species and aetiologies. In 2005 and 2006, a combined total of 995 garden birds of 42 species were examined, of which Fringillidae species accounted for 64% of submissions.

Fresh carcasses were submitted by post or were hand-delivered and were refrigerated at 4°C and examined fresh within 48 hours of submission where possible, or were frozen at −20°C on submission and examined at a later date. Each submitted carcass was assigned a unique post mortem reference code and the species, age, sex and body weight were recorded for each bird examined. Systematic external and internal examinations of body systems were performed and any gross lesions described. Where indicated, and where the state of carcass decomposition permitted, samples were taken for microbiological, parasitological and histopathological investigations.

Liver and contents of the mid-small intestinal loop were sampled aseptically from the majority of cases, as were any lesions found, and were examined for the presence of pathogenic bacteria. Briefly, liver was plated directly onto the following media: Colombia blood agar supplemented with 5% horse or sheep blood (CBA)(QCM laboratories, UK or E and O laboratories, UK), incubated under aerobic conditions, and Chocolate blood agar (CCBA) (QCM laboratories), incubated under 5–10% CO_2_ conditions. Small intestinal contents were plated directly onto the following media: (1) Xylose-Lysine Deoxycholate (XLD) agar (QCM laboratories), or MacConkey agar without salt (E&O laboratories) and Brilliant green agar (E&O laboratories), incubated under aerobic conditions; (2) CBA, incubated under aerobic conditions; (3) Campylobacter Blood Free Selective medium (modified CCDA-Preston) (QCM laboratories), incubated under microaerophilic conditions, and (4) immersed into selenite Salmonella-selective enrichment broth (QCM laboratories or E&O laboratories) under aerobic conditions for 24 h followed by subculture onto XLD agar aerobically. At the Institute of Zoology, liver also was plated onto CBA and incubated anaerobically. The same bacteriology protocol was used for examination of necrotic ingluvitis lesions as for the intestinal contents with the exception of the modified CCDA-Preston media. Bacterial isolates were identified using colony and Gram's staining morphology, followed by biochemical properties which were determined using the API biochemical test strip method (API-BioMerieux, Marcy l'Etoile, France).

In addition, oesophageal lesions (circa 5 mm^3^) from cases with necrotic ingluvitis were incubated at 30°C in Trichomonas Media No. 2. (Oxoid, UK) and screened for motile trichomonads at 24, 48, 72 hrs and 5 days. Wet mount preparations of small intestinal contents were examined in a subset of cases for evidence of nematode, cestode and protozoan parasites.

Samples from a range of organs (including brain, gizzard, heart, kidney, liver, lung, pectoral muscle, small intestine, spleen, trachea and any diseased tissues), were fixed in neutral-buffered 10% formalin and processed for histopathological examination using routine methods. Duplicate samples of organs and diseased tissues were stored frozen at −20°C or −80°C for future analyses.

A combination of morphological and molecular techniques was used to identify the trichomonad species. Giemsa-stained preparations of trichomonad cultures were examined using light microscopy to assess parasite morphology. These were prepared by placing a drop of active trichomonas culture onto a standard glass microscope slide; this was then air dried, alcohol-fixed and stained using routine methods. Transmission and scanning electron microscopy was performed on trichomonad cultures fixed in 2.5% buffered gluteraldehyde and post-fixed in 1% osmium tetroxide (VWR, UK) at the University College Medical School, Royal Free Campus, using Philips 201 and 501 microscopes.

DNA was extracted from frozen/thawed necrotic ingluvitis lesions collected from finches using the Biosprint 15 DNA Blood Kit (Qiagen, UK) for purification of DNA from tissue according to the manufacturer's instructions. DNA was extracted from trichomonad cultures using the same technique. PCR was used to amplify the ITS1/5.8S/ITS2 ribosomal region using published TFR1 and TFR2 primers [30] with an adapted protocol. Briefly, PCR reactions were run with 3 µL of 10X PCR buffer (Qiagen), 3 µL of 25 mM MgCl_2_ (Qiagen), 0.5 µL of 5 U/µL HotStar Taq Plus DNA Polymerase (Qiagen), 2 µL template DNA, 0.4 µL of 100 mM dNTP mix (Bioline, UK), 3 µL of 10 µM forward and reverse primer and molecular grade water to complete the 50 µL per reaction. Oligonucleotide primers were supplied by Operon Biotechnologies, Germany. After an initial 15 min denaturation at 94°C, 35 cycles of 94°C for 1 min, 65°C for 30 sec and 72°C for 1 min were carried out, followed by a 5 min extension at 72°C using a thermal cycler (Tec-571, Techne, UK). Each PCR run contained a negative control of water and a positive control of purified trichomonad DNA obtained from parasites cultured from an affected greenfinch found dead as part of this study.

The PCR products, consisting of a clear single band, were visualised under UV light after ethidium bromide staining of a 1% agarose gel and the expected product size (circa 400 bps) was confirmed using Ready-Load 100 bp DNA ladder (Invitrogen, UK). PCR products were purified using the QIAquick PCR purification kit (Qiagen) and submitted for sequencing at the John Innes Genome Laboratory, UK, using the Applied Biosystems 3730xl with POP7 polymer and the TFR1 and TFR2 primers. Chromatograph profiles were inspected using Chromas 2 software (www.synthesisgene.com). Sequences from the forward TFR1 primer and the reverse complement of the TFR2 primer PCR product were aligned in both directions for each sample using MEGA 4.1 software and ClustalW (www.megasoftware.net). Sequences were compared with available gene sequences within NCBI Genbank using the BLAST search function to determine species identification within the Trichomonadidae.

### Molecular detection of Trichomonas infection

A nested PCR protocol was designed to increase the sensitivity of detection of trichomonad parasite DNA in template DNA extracted from lesions sampled post mortem and to provide a diagnostic tool for cases where autolysed carcass condition precluded *Trichomonas* sp. culture. Trichomonad small subunit (SSU) rRNA primers (forward -TACTTGGTTGATCCTGCC and reverse - TCACCTACCGTTACCTTG) from [43] were used for the first reaction. The PCR product nucleotide sequence was obtained from a pure trichomonad culture obtained from an affected greenfinch. Nested primers, TN3 forward (ATAGGACTGCAAAGCCGAGA) and TN4 reverse (TGATTTCACCGAGTCATCCA), were then designed using Prime3 online software [44]. Primers were supplied by Eurofins MWG Operon (UK).

The first stage used 2 µL of 10X PCR buffer (Qiagen), 0.1 µL of 5 U/µL HotStar Taq Plus DNA Polymerase (Qiagen), 2 µL template DNA, 0.4 µL of 10 mM each dNTP mix (Qiagen), 0.4 µL of 100 µM forward and reverse primer and molecular grade water to complete the 20 µL reaction. After an initial 5 min denaturation at 94°C, 40 cycles of 94°C for 1 min, 55°C for 1 min and 72°C for 2 mins were carried out, followed by a 5 min extension at 72°C using a thermal cycler (GeneAmp PCR System 9700, Applied Biosystems, UK).

The reaction mixture for the second amplification was the same as for the first, except for the use of the nested primers. The amplification mix comprised 19 µl of mix and 1 µl of PCR product template from the first amplification round. After an initial 5 min denaturation at 94°C, 35 cycles of 94°C for 45 sec, 50°C for 45 sec and 72°C for 45 sec were carried out, followed by a 7 min extension at 72°C. Each amplification contained a negative control, consisting of water and a positive control of purified DNA obtained from cultured trichomonad parasites from a greenfinch. Amplified PCR products were visualised under UV light after ethidium bromide staining of a 3% agarose gel and the expected product size (circa 200 bps) was confirmed using Easy Ladder I (Bioline). PCR products were submitted for sequencing at Cogenics (UK) using the ABI 3730 xl platform with the TN3 forward and TN4 reverse primers. Chromatograph profiles were inspected using Chromas 2 software. The sequence from the forward TN3 primer and the reverse complement of the TN4 primer PCR product were aligned in both directions for each sample using MEGA 4.1 software and ClustalW. Sequences were compared with available gene sequences within NCBI Genbank using the BLAST search function.

Reliable discrimination between necrotic ingluvitis due to salmonellosis or trichomonosis in finches is not possible based on gross examination alone. In order to evaluate whether our nested PCR cross-reacted non-specifically with *Salmonella* Typhimurium Definitive Type (DT)40 and DT56 variant (v), lesions from 56 greenfinches and six chaffinches confirmed to have died due to salmonellosis by microbiological examination in 2005 and 2006 (Lawson, *unpublished data*) were screened. The majority of cases were negative on nested PCR (54/62 cases) and all cases examined during the study period 1 April 2006 to 30 September 2006 were negative. This indicates that 8 cases examined outside the study period might have had concurrent infection with *Trichomonas* sp. and *Salmonella* sp.. Carcass condition precluded parasite culture in all but one of these cases, but this case yielded trichomonad parasites confirming the existence of dual infection. These findings suggest that the nested PCR does not cross react non-specifically with *Salmonella* Typhimurium DT40 and DT56(v) and can be used within our case definition for the diagnosis of trichomonosis in finches.

### Case definition

Cases of trichomonosis were diagnosed on the basis of the presence of necrotic ingluvitis lesions in combination with positive culture of motile trichomonads and/or positive nested PCR amplification. Salmonellosis also causes necrotic ingluvitis and this was diagnosed by lesions being positive for *Salmonella* sp. on culture. No cases of dual infection were identified during the study period.

### Geographical and temporal distribution of the epidemic

Garden Bird Watch participants (c. 9000) record the presence of a range of bird and other wildlife species encountered each week throughout the year; a subset (c. 30%) submit actual counts of each species seen using an online recording form. Participants maintain a consistent level of observational effort from one week to the next; data from weeks that are under- or over-observed are discarded. Variation in observer effort and competence is inevitable, but this can be controlled for by introducing a site effect into the models used to examine the data [40]. Almost all the participants provide food of some kind (the range of food provided for garden birds is also recorded on a weekly basis) for wild birds and feeding stations are generally the focal point of the study areas. The subset of participants that took part in GBH*i* surveyed all or part of their garden systematically in a consistent manner each week to record the number, and putative cause, of dead or sick birds found. The clinical signs of ill health in birds affected by trichomonosis typically included non-specific malaise, although dysphagia was noted in a large proportion of reported incidents. This contrasts with the clearly recognisable external signs of conjunctivitis caused by *Mycoplasma gallisepticum* in the house finch and which were used to monitor spatial spread of mycoplasmosis [20].

In order to evaluate the geographical distribution of the trichomonosis epidemic, data from the opportunistic and systematic reporting schemes were examined for the period between 1 April and 30 September 2006. This interval was selected to minimise the likelihood of confounding these data with mortality due to salmonellosis, outbreaks of which occur during the winter months and which also result in non-specific signs of malaise in finches. Previous studies of salmonellosis in Great Britain [45], continental Europe [46] and North America [47] along with our own observations on salmonellosis in passerines over the period 1995–2008 (Lawson and Cunningham, *unpublished data*), have shown the disease to be seasonal, occurring almost exclusively within the period 1^st^ October to 31^st^ March.

### Incident definition

As resources and logistics did not allow all dead birds found to be submitted for post mortem examination, we established specific criteria for determining if a mortality incident should be classified as likely being due to trichomonosis. For the purposes of this analysis, a trichomonosis incident was defined if, within the six month period of the study (between 1 April and 30 September 2006), mortality included two or more dead finches (greenfinch or chaffinch), one or more sick finch(es) with typical signs of disease, or if trichomonosis was confirmed post mortem. To give a measure of incidence for the opportunistic reports, we expressed the total number of trichomonosis incidents reported in each county per thousand households according to the 2001 UK National Census [48].

### Changes in bird abundance

Each week, GBW participants recorded the presence of each bird species in their garden and, optionally, the peak number of birds counted each week [22]. For this analysis we used the 828 gardens for which data were submitted in every week from January 2005 to June 2007 in England south of 53.5°N (see below). We modelled bird abundance as the proportion of gardens in which the species was present (‘reporting rate’). We modelled reporting rates using generalised additive or linear mixed models, fitted using the gamm function in package *mgcv* for R 2.6.0 [49], [50].

To exclude the possibility that changes in other environmental factors, such as climate, between years might have caused changes in greenfinch numbers, we also modelled reporting rates of chaffinch, which has a similar body size and ecology to the greenfinch, and was the second most frequently affected species in which trichomonosis was recorded, and of dunnock, which also feeds around garden feeders [51] but in which trichomonosis was rarely recorded (only three cases diagnosed in 2005 and 2006 combined). Both species show similar patterns of spatial and temporal abundance in gardens to the greenfinch [22]. We predicted that, if trichomonosis was responsible for changes in numbers, chaffinch should show an identifiable, but less marked, response and dunnock should show little response; if other factors, such as climatic factors or a change in resource availability, were involved then the response among the three species should be similar.

Abundance of birds in gardens varies seasonally in a non-linear fashion, with peak numbers occurring in late winter or spring and lowest counts in late summer/autumn, when many birds are moulting [22], [35]. To model this variation we fitted a generalised additive model to the reporting rate as a function of week number (1–7 Jan = 1 to 25–31 Dec = 52) in the form of a thin-plate regression spline using gamm with a binomial error structure and logit link function [50]. We used the default level of smoothing, as increasing the potential for smoothing (by changing the basis, k) did not alter the results materially. To account for variation among gardens in the probability of birds being present and observer ability we included garden identifier as a random-effect term. Preliminary models including garden identifier as a fixed effect showed the distribution of these effects, as for other large-scale schemes run by BTO, were indeed approximately normally distributed. The weekly records of bird presence are likely to exhibit serial auto-correlation, both temporally and spatially. We initially fitted models with no temporal or spatial auto-correlation and while temporal correlation among the residuals was strong, spatial correlations (analysed using the correlog function in library ncf [52]), though statistically significant, were typically weak (r≤0.05). This may reflect the fact that distance between gardens was generally much greater than the ambit of individual foraging flocks and it is unlikely the provisioning behaviour in any one sample garden influenced the behaviour in neighbouring sample gardens. We therefore included an AR1 auto-regressive correlation structure (with weeks numbered consecutively through the entire period) to account for the correlation between records in consecutive weeks; the degree of auto-correlation was assumed to be the same for each garden.

To quantify impacts on the breeding season following the epidemic of trichomonosis in autumn 2006, we constructed a model to compare reporting rate at the start of the 2007 breeding season (weeks 13–21, 26 March–28 May) with the average reporting rate for the same period in the previous two years (which, as far as could be determined, were typical). We restricted the analysis to the previous two years to avoid potential confounding effects of long-term trends in bird numbers and reporting rates. As we were considering a relatively short time-span within a year, it was not necessary to fit a smoothed term of week; rather we modelled reporting rate as a function of garden size, week (and its square to account for any non-linearity) and a two-level dummy variable, year, with weeks in 2005/06 having year = 0 and those in 2007 year = 1. The estimate of this latter term then gives the change in reporting rate in 2007 relative to that in the previous two years. As before, we included garden identifier as a random effect and fitted an auto-correlated binomial error structure.

Mortality due to trichomonosis varied spatially throughout the country, so we defined three regions representing areas of High, Intermediate and Low incidence of trichomonosis, based on the results of opportunistic sampling. We restricted these analyses to England south of a line from the Mersey to the Humber (approx. 53° 30′N) as it is in this region that gardens participating in GBW are most representative of the landscape as a whole (for example upland areas, where there tend to be few GBW sites, are relatively limited in extent). Greenfinch, chaffinch and dunnock populations occur widely across this region, being observed in 70–80% of gardens in the GBW scheme. For convenience, we defined these three regions in terms of administrative county boundaries with areas of High (Cheshire, Derbyshire, Gloucestershire, Herefordshire, Leicestershire, Shropshire, Staffordshire, Warwickshire and the West Midlands), Intermediate (Bedfordshire, Buckinghamshire Cambridgeshire, Lincolnshire, Northamptonshire, Nottinghamshire, Oxfordshire, South Yorkshire and Wiltshire) and Low (Berkshire, Essex, Hampshire, Hertfordshire, Greater London, Kent, Norfolk, Suffolk, Surrey and Sussex) incidence of trichomonosis. To test for differences in the change in reporting rate in spring 2007 among areas, we included an interaction term between area and the dummy year variable described above.

Numbers of birds present across Britain during the breeding season are monitored using line-transect counts in a sample of c. 3,000 randomly selected 1×1 km squares by the Breeding Bird Survey (BBS) [23]. BBS transects are undertaken in all habitats, rather than being restricted to gardens as with the GBW scheme. An index of relative abundance based on a generalized linear Poisson model with categorical site and year fixed-effects is produced annually; we obtained indices for the three county groupings to measure the relative change in breeding population between 2006 and 2007 in each region.
